# Denialism: How Irrational Thinking Hinders Scientific Progress, Harms the Planet, and Threatens Our Lives

**DOI:** 10.3201/eid1604.091710

**Published:** 2010-04

**Authors:** Tara C. Smith

**Affiliations:** University of Iowa, Iowa City, Iowa, USA

**Keywords:** Denialism, vaccines, biotechnology, organic food, global warming, HIV/AIDS, book review

Vaccines remain one of our most critical public health interventions, yet an increasingly vocal group of antivaccinationists call vaccines toxic and attribute numerous conditions, such as autism, to vaccination despite evidence to the contrary. Former Playboy model Jenny McCarthy is viewed as a respected source of information, whereas pediatric infectious diseases researcher Paul Offit receives death threats for his scientific contributions. Journalist Michael Specter describes this phenomenon in Denialism: How Irrational Thinking Hinders Scientific Progress, Harms the Planet, and Threatens Our Lives.

Spector defines denialism as “denial writ large—when an entire segment of society, often struggling with the trauma of change, turns away from reality in favor of a more comfortable lie.” Denialism describes this rejection of fact-based reality in 6 different areas related to health and medicine, beginning with the mistrust of pharmaceutical companies and their products. He also examines the dismissal of racial differences in medical research, the organic food movement as a form of denialism, and the obsession with vitamins and complementary alternative medicine despite evidence that these modalities do not work or may even be harmful. He ends with a look to the future: creation of life itself and why we must overcome denialism and embrace change to continue advancing as a society or risk the survival of our species.

This book is sure to cause some controversy. In many circles, the term denialism is still linked to rejection of the Holocaust, and any implication that parents’ refusal of vaccination or the philosophy of choosing organic-only food is akin to dismissal of one of humanity’s great atrocities surely will make some readers dismiss Specter’s arguments. Likewise, although Specter supports industry, he is pragmatic and does not put himself in a cheerleader role, instead acknowledging corporate culpability in driving and perpetuating denialism: “Corporations, wrapping themselves in the mantle of progress but all too often propelled by greed, have done more than religion or even Luddism to inflame denialists and raise doubts about the objectivity of science.” Specter realizes that industry, and scientists, have lost the public’s trust, and this broken relationship needs to be fixed through additional communication to the public by scientists and science writers, open debates about the future of scientific progress and implications of emerging technologies, and improvements in education.

Persons picking up this book may be surprised by the lack of discussion about some prominent topics of science denialism, including evolution and global warming. Likewise, Specter does not discuss HIV/AIDS denial, a topic he has covered previously in The New Yorker (*1*). Referring to the latter, Specter notes that Holocaust and HIV/AIDS denialists are “… intensively destructive—even homicidal—but they don’t represent conventional thought and they never will. This new kind of denialism is less sinister but more pervasive than that.” Furthermore, the types of denialism Specter describes cut across political and religious divisions to combine fear and uncertainty in a manner that makes them contagious. Indeed, a central tenet of denialism is that “fear is more infectious than any virus” and, like infection, needs to be addressed and, ideally, prevented through healthy skepticism.

Spector notes that “Denialism must be defeated. There is simply too much at stake to accept any other outcome.” Its defeat is a tall order, but an imperative one if science is truly to be restored to its rightful place. Specter’s book is a good starting place for any scientist or layman interested in delving into this phenomenon.
